# Markedly Elevated Carbamazepine-10,11-epoxide/Carbamazepine Ratio in a Fatal Carbamazepine Ingestion

**DOI:** 10.1155/2015/369707

**Published:** 2015-10-13

**Authors:** Jason L. Russell, Henry A. Spiller, Daniel D. Baker

**Affiliations:** ^1^The Ohio State University Wexner Medical Center, Columbus, OH 43210, USA; ^2^Nationwide Children's Hospital, Columbus, OH 43205, USA; ^3^Central Ohio Poison Center, Columbus, OH 43205, USA; ^4^Franklin County Coroner's Office, Columbus, OH 43201, USA

## Abstract

Carbamazepine is a widely used anticonvulsant. Its metabolite, carbamazepine-10,11-epoxide, has been found to display similar anticonvulsant and neurotoxic properties. While the ratio of parent to metabolite concentration varies significantly, at therapeutic doses the epoxide concentration is generally about 20% of the parent. We report a case of fatal carbamazepine overdose in which the epoxide metabolite concentration was found to be 450% higher than the parent compound, suggesting a potential role for metabolite quantification in severe toxicity.

## 1. Introduction

The toxicity from carbamazepine (CBZ) overdose has been well described and includes cardiac dysrhythmias, hypotension, coma, respiratory depression, and seizures [1]. Generally serum levels of CBZ are used to assess or predict toxicity: with serum levels < 30 mcg associated with mild to moderate toxicity and serum levels > 40 mcg/mL associated with major toxicity symptoms (apnea, seizures, and coma) [2–4]. However these serum CBZ levels do not take into account the major active metabolite carbamazepine-10,11-epoide (CBZE) which shares roughly equal anticonvulsant and neurotoxic effects with the parent drug CBZ [5, 6]. Serum concentrations of the metabolite CBZE generally range from 15 to 40 percent of the parent drug in therapeutic concentrations and can rise to 50 to 80% of CBZ concentration after overdose [7–9]. A number of drugs such as quetiapine, phenytoin, and valproic acid have been reported to alter CBZ metabolism, significantly increasing serum levels of CBZE to as high as 90% to 150% of the parent drug [7, 10]. An additional important feature of CBZE is that, unlike the parent drug CBZ, as serum CBZE levels rise protein binding is saturated and the amount of free CBZE increases, with subsequent increased availability for CNS toxicity [11].

We report a case of profoundly elevated CBZE concentrations in the presence of moderately elevated CBZ concentration after a CBZ overdose and suggest a role for CBZE measurement in the management of severe CBZ toxicity.

## 2. Case Report

A 21-year-old female weighing approximately 59 kilograms presented to a community-based Emergency Department for assessment of a recent intentional ingestion of multiple medications. According to her family, the patient had ingested as much as 17.8 grams of carbamazepine, 4.5 grams of hydroxyzine, 14.4 grams of ibuprofen, 1.2 grams of fluoxetine, and an unknown amount of lamotrigine. The ingestion occurred approximately 1 hour prior to presentation. Upon presentation the patient was described as having an unsteady gait, slurred speech, confusion, tachycardia, hypertension, and suicidal ideation and was uncooperative with hospital personnel.

In the ED her immediate management consisted of benzodiazepines for agitation and IV fluid administration. Vital signs upon arrival included a blood pressure of 155/117 and heart rate of 124. No initial temperature was recorded. Laboratory analysis was recommended and the following pertinent positive and negative results were recorded: salicylate < 1 mg/dL, acetaminophen < 15.0 mcg/mL, EtOH undetectable, CPK 462 U/L, Lactic Acid 3.0 mmol/L, and Bicarbonate 17 mmol/L. The patient's initial carbamazepine level, drawn between 1 and 2 hours after ingestion, was 24 mcg/mL [4.0–12.0 mcg/mL]. An EKG performed shortly after arrival demonstrated the following intervals: heart rate 124, QTc 445 msec, and QRS 78 msec. Subsequent repeat EKG studies revealed a gradually increasing QTc measurement. While in the ED the longest QTc measured was 479 msec. While in the Emergency Department the patient experienced multiple episodes of tonic-clonic seizure-like activity lasting 15–20 seconds. Transfer to a tertiary care facility was arranged. Prior to transport the patient was intubated due to altered mental status and for airway protection. Following intubation the patient experienced worsening seizure frequency and duration.

Over the course of the following 6 days the patient's clinical course continued to worsen. Concern was raised for potential serotonin syndrome due the presence of uncontrollable seizures. Cyproheptadine therapy was initiated by the tertiary care facility via NG tube and was continued throughout her treatment. She continued to receive benzodiazepines as well as propofol for control of recurrent seizure activity. Due to persistent seizure activity CPK remained elevated with a peak of 1262 IU/L. During this period renal function remained normal with a BUN ranging from 7 to 10 mg/dL and creatinine ranging from 0.74 to 0.86 mg/dL.

The patient was monitored closely for electrocardiographic abnormalities; though her QRS remained less than 100 msec throughout, her QTc prolonged to as much as 663 msec. This figure was significantly complicated by numerous coadministered medications and electrolyte abnormalities.

The patient developed ARDS on hospital day #3. Despite aggressive resuscitation the patient expired after a series of cardiac arrests on hospital day #7.

Serial carbamazepine levels were drawn during the hospital stay. Please see Table 1 for details. The patient's CBZ level declined during the first 48 hours of her stay, only to increase significantly on hospital days three through six.

Following the patient's death on hospital day #7 her body was transferred to the county coroner's office for postmortem examination. The cause of death was determined to be complications of acute intoxication by the combined effects of carbamazepine, carbamazepine-10,11-epoxide, and topiramate. Topiramate was a previously unknown intoxicant in this case.

## 3. Methodology and Results of Postmortem Analysis

Gross examination did not reveal any significant or unexpected abnormalities given the clinical course. Forensic laboratory reported serum results are summarized in Table 2. Drugs were extracted from 1.0 mL serum using Clean Screen CSDAU206 hydrophobic/cation exchange solid phase extraction columns (UCT, Inc., Bristol, PA). Drugs were eluted from the columns in a two-step sequential elution, first liberating acidic/neutral drugs then basic drugs. The separate extracts were concentrated and reconstituted in 500-microliter acetonitrile and 100-microliter acetonitrile, respectively. The extracts were analyzed using full scan gas chromatography/mass spectroscopy with electron ionization. Carbamazepine/metabolites, topiramate, and phenytoin were identified in the acidic/neutral extract analysis, whereas lamotrigine, fluoxetine, and hydroxyzine/metabolites were identified in the basic extract analysis. Carbamazepine, carbamazepine-10,11-epoxide, and lamotrigine serum quantitation was performed by NMS Labs, Willow Grove, PA, using liquid chromatography with UV/VIS detection measured at 215 nm. Topiramate serum level was additionally determined by NMS Labs, using liquid chromatography tandem mass spectroscopy. Reference ranges were determined via NMS laboratory standards.

In addition to these studies vitreous electrolytes were performed and resulted as follows: sodium 152 mmol/L, potassium 16 mmol/L, chloride 150 mmol/L, calcium 1.5 mmol/L, magnesium 0.76 mmol/L, glucose 28 mmol/L, lactate 17 mmol/L, urea nitrogen 22 mg/dL, and creatinine 3.2 mg/dL.

## 4. Discussion

The antemortem serum sample drawn on hospital day #3 reveals a 4.5/1 CBZE/CBZ ratio. This is dramatically higher than what has previously been reported. At steady state the previously reported value for CBZE levels in healthy patients was 10%–50% of the serum CBZ concentration [12]. de Groot et al. reported a ratio of 1.58 ± 0.16 in a patient with concomitant valproic acid and phenytoin use [10]. So et al. reported on a group of 5 adults in whom seizure control had deteriorated despite multidrug therapy [8]. In those individuals the addition or increase of another drug, most commonly valproic acid, elevated CBZE/CBZ ratios [8]. Adjustment of the patient's medication by decreasing the amount of administered CBZ resulted in a decrease in the CBZE/CBZ ratio and increased seizure control [8]. This suggests that an increased CBZE/CBZ ratio, as well as increased serum CBZE, contributes to seizure frequency and intensity.

By hospital day #6 the patient's CBZ level had elevated to nearly the same peak level as recorded in the first 48 hours of toxicity. This may have been due to retained drug in the gut, impaired hepatic metabolism, drug-drug interaction, or other factors. As this finding may represent prolonged absorption this patient may have benefited from early GI decontamination.

Several features may have played a role in the elevated CBZE concentration and CBZ/CBZE ratio. Carbamazepine is metabolized primarily by CYP3A4 in humans. Induction of CYP3A isoforms by other antiepileptic drugs has been shown to increase the metabolism of CBZ to CBZE in animal models [13]. Topiramate, found in toxic concentrations in this patient, has been shown to induce CYP3A4 in vitro [14]. In that study the degree of CYP3A4 induction was increased with higher concentrations of topiramate. The coingestion of both drugs in toxic amounts may be at least partially responsible for the markedly high CBZE/CBZ ratio, though individual genetic factors may be involved as well. Lamotrigine was found in our patient. However, previous studies have shown that lamotrigine exhibited no effect on CBZ or CBZE concentrations [15, 16]. Renal clearance may affect CBE/CBZE ratios; however in our patient renal function appeared normal when the 4.5/1 CBZ/CBZE ratio was recorded on hospital day 3 [17]. Propofol is known to produce mild inhibition of cyp3A4 but in therapeutic concentrations is believed to unlikely have a clinically significant effect [18].

The CBZE/CBZ ratio observed in this patient highlights a potentially significant contribution CBZE quantification may play in the large overdose. Routine measurement of CBZE serum concentration may not be necessary in routine settings [19]. However if observed clinical effects are in excess of what would normally be expected at a given CBZ serum concentration, CBZE quantification may be valuable. As demonstrated previously, both CBZ and CBZE are effectively cleared via hemodialysis [20]. In this case the falling CBZ level may have been falsely reassuring despite ongoing severe symptoms. A CBZE level may have encouraged more aggressive management.

## Figures and Tables

**Table 1:** Serial serum carbamazepine levels drawn during hospital stay.

Date/time of laboratory collection	Serum carbamazepine level [4.0–12.0 mcg/mL]
Arrival	24.0 mcg/mL
Hospital day #1, evening	19.5 mcg/mL
Hospital day #1, night	18.7 mcg/mL
Hospital day #2, morning	19.3 mcg/mL
Hospital day #3, morning	10.5 mcg/mL
Hospital day #4, midday	12.8 mcg/mL
Hospital day #4, night	14.3 mcg/mL
Hospital day #5, morning	16.7 mcg/mL
Hospital day #5, evening	18.0 mcg/mL
Hospital day #5, night	19.9 mcg/mL
Hospital day #6, morning	20.4 mcg/mL

Serum carbamazepine analysis performed on a Beckman Coulter AU5800 Clinical Chemistry.

System using Beckman Coulter *Emit* 2000 Carbamazepine Assay.

**Table 2:** Antemortem serum drawn on hospital day #3, morning (processed postmortem).

Analytical methodology	Analyte	Serum result
GC/MS-EI full scan	Caffeine	Positive
HPLC-UV/VIS	Carbamazepine	12 mcg/mL
HPLC-UV/VIS	Carbamazepine-10,11-epoxide	54 mcg/mL
GC/MS-EI full scan	Iminostilbene	Positive
HPLC-UV/VIS	Lamotrigine	5.7 mcg/mL
GC/MS-EI full scan	Fluoxetine	0.3 mcg/mL
GC/MS-EI full scan	Norfluoxetine	Not detected
GC/MS-EI full scan (TMS derivative)	Hydroxyzine	Trace (less than 0.1 mcg/mL)
GC/MS-EI full scan	Hydroxyzine metabolite	Positive
GC/MS-EI full scan	Cetirizine	Positive
GC/MS-EI full scan	Laudanosine (atracurium metabolite, administered in hospital)	Not detected
LC/MS/MS-ESI	Topiramate	28 mcg/mL
GC/MS-EI full scan	Phenytoin (administered in hospital)	Positive
